# AIDS-related deaths in Turkey between 2009 and 2018

**DOI:** 10.1017/S0950268821001539

**Published:** 2021-07-02

**Authors:** Ayse Gulsen Teker

**Affiliations:** Marmara University School of Medicine Department of Public Health, Maltepe-Istanbul, Turkey

**Keywords:** AIDS, death, epidemiology, HIV, Turkey

## Abstract

The prevalence of human immunodeficiency virus/acquired immunodeficiency syndrome (HIV/AIDS) is increasing day by day in the region, including Turkey. The study aimed to examine AIDS-related deaths in Turkey between 2009 and 2018 according to the national death registration system records. In this descriptive study, data on AIDS-related deaths were obtained from the Turkish Statistical Institute. The data consist of the cause of death codes, year of death, age and gender. Findings were presented using numbers and percentages. Seven hundred twenty-one AIDS-related deaths were reported in Turkey between 2009 and 2018. AIDS-related deaths in Turkey increased more than twice at the end of 10 years. The male/female death ratio is 4.5. Deaths under the age of 15 were 4.2% in total; however, they were increased to 10.2% in 2018. AIDS-related deaths are decreasing in the world but increasing in Turkey. The data from the Ministry of Health do not match the data of the national death registration system. Establishing a strong and accurate HIV/AIDS reporting system and identifying the causes and risk groups of this increase in AIDS-related deaths are critical.

## Introduction

A total of 75.7 million people have been diagnosed with *human immunodeficiency virus* (HIV) infection since the report of the first case in 1981, and 32.7 million people have died due to acquired immune deficiency syndrome (AIDS) [1]. The first case in Turkey was diagnosed in 1985. After this date, 19 748 people were diagnosed with HIV infection in Turkey [2]. The number of new cases gradually decreased in the world after the peak observed in 1997 with 2.2 million new cases, and the number of new cases was 1.7 million in 2018. There is also a decrease in the rate of new HIV cases throughout Europe. In contrast, there has been an increase in some countries, including Turkey, according to the report prepared jointly by European Center for Disease Prevention and Control (ECDC) and World Health Organization Regional Office for Europe [3]. The number of new cases increased by 29% in Eastern Europe and Central Asia Region of UNAIDS (The Joint United Nations Program on HIV/AIDS), including Turkey, in 2018, contrary to the decrease in the world. The number of AIDS-related deaths also tended to decrease in the world, similar to the number of new cases and was reported to be 770 000 in 2018, reduced by 33% compared to 2010. However, AIDS-related deaths in Eastern Europe and Central Asia Region, where Turkey is also located, increased by 5% in the same period [4].

Notification of HIV cases is mandatory since 1985, when the first case was seen in Turkey. Since 1994, case reports have been coded due to patient privacy [2]. Deaths in Turkey are recorded by the Turkish Statistical Institute (TURKSTAT), which is a separate institution from the Ministry of Health. TURKSTAT records every death seen in the country. The causes of death are coded according to the Tenth Revision of the International Statistical Classification of Diseases and Related Health Problems (ICD-10) classification [5].

Effective information gathering, recording and sharing are critical in the fight against HIV/AIDS. Recording, examining and presenting AIDS-related deaths are also important. The study aimed to examine AIDS-related deaths in Turkey between 2009 and 2018, according to TURKSTAT data.

## Methods

### Study design

#### Descriptive study

Data for this descriptive study were obtained from the Turkish Statistical Institute (TURKSTAT). TURKSTAT provided information on all AIDS-related deaths in 2009 and 2018 in Excel files and without sharing any personal data upon researchers' requests. The data consist of the cause of death codes, year of death, age and gender. According to TURKSTAT data, AIDS-related deaths were coded as B21, B22, B23, B24 and R75 in ICD-10 (Table 1).

**Table 1.** HIV/AIDS codes according to the international classification of diseases 10th revision (icd 10) version 2010

Code	Definition
B20	HIV disease, resulting in infectious and parasitic diseases
B21	HIV disease, resulting in malignant neoplasms
B22	HIV disease resulting in other specified diseases
B23	HIV disease resulting in other conditions
B24	Unspecified HIV disease
R75	Laboratory evidence of HIV disease

### Statistics

Age and gender characteristics were evaluated according to the years of the deaths. Findings were presented using numbers and percentages.

## Results

A total of 721 people died due to AIDS-related causes between 2009 and 2018. Both male and female deaths have increased, and total deaths have more than doubled. Of AIDS-related deaths, 590 (81.8%) were male, and 131 (18.2%) were female. The male/female ratio was 4.5. In the whole 10-year period, male deaths were more than female deaths. Table 2 presents the number of deaths of women and men due to AIDS-related causes by years (Table 2).

**Table 2.** Distribution of AIDS-related deaths by gender in Turkey between 2009 and 2018

Years	Gender		
	Male *n* (%)	Female *n* (%)	Total *n* (%)
2009	39 (86.7)	6 (13.3)	45 (100.0)
2010	43 (79.6)	11 (20.4)	54 (100.0)
2011	38 (86.4)	6 (13.6)	44 (100.0)
2012	54 (87.1)	8 (12.9)	62 (100.0)
2013	56 (75.7)	18 (24.3)	74 (100.0)
2014	67 (84.8)	12 (15.2)	79 (100.0)
2015	71 (77.2)	21 (22.8)	92 (100.0)
2016	75 (88.2)	10 (11.8)	85 (100.0)
2017	69 (78.4)	19 (21.6)	88 (100.0)
2018	78 (79.6)	20 (20.4)	98 (100.0)
Total	590 (81.8)	131 (18.2)	721 (100.0)

It was observed that the number of deaths in the age groups occurred most frequently in the 35–44 and 45–54 age groups. Of 721 deaths, 175 (24.3%) were in the 35–44 age group, and 205 (28.4%) were in the 45–54 age group. Deaths in the group under the age of 15 constituted 4.2% of the total, but deaths in this group accounted for 10.2% of the deaths in 2018. Table 3 shows the distribution of AIDS-related deaths by years in age groups (Table 3).

**Table 3.** Distribution of AIDS-related deaths by age groups in Turkey between 2009 and 2018

	Age groups									
Years	0–14	15–24	25–34	35–44	45–54	55–64	65–74	75–84	+85	Total
2009	1 (2.2)	2 (4.4)	10 (22.2)	10 (22.2)	13 (28.9)	2 (4.4)	5 (11.1)	2 (4.4)	0 (0.0)	45 (100.0)
2010	4 (7.4)	0 (0.0)	6 (11.1)	15 (27.8)	15 (27.8)	5 (9.3)	6 (11.1)	2 (3.7)	1 (1.9)	54 (100.0)
2011	2 (4.5)	1 (2.3)	4 (9.1)	14 (31.8)	14 (31.8)	6 (13.6)	3 (6.8)	0 (0.0)	0 (0.0)	44 (100.0)
2012	2 (3.2)	0 (0.0)	7 (11.3)	18 (29.0)	19 (30.6)	4 (6.5)	9 (14.5)	2 (3.2)	1 (1.6)	62 (100.0)
2013	0 (0.0)	3 (4.1)	13 (17.6)	17 (23.0)	23 (31.1)	10 (13.5)	6 (8.1)	2 (2.7)	0 (0.0)	74 (100.0)
2014	2 (2.5)	1 (1.3)	19 (24.1)	17 (21.5)	20 (25.3)	14 (17.7)	3 (3.8)	2 (2.5)	1 (1.3)	79 (100.0)
2015	1 (1.1)	1 (1.1)	12 (13.0)	22 (23.9)	25 (27.2)	17 (18.5)	7 (7.6)	6 (6.5)	1 (1.1)	92 (100.0)
2016	4 (4.7)	0 (0.0)	14 (16.5)	18 (21.2)	28 (32.9)	7 (8.2)	9 (10.6)	4 (4.7)	1 (1.2)	85 (100.0)
2017	4 (4.5)	2 (2.3)	16 (18.2)	21 (23.9)	18 (20.5)	15 (17.0)	6 (6.8)	6 (6.8)	0 (0.0)	88 (100.0)
2018	10 (10.2)	2 (2.0)	10 (10.2)	23 (23.5)	30 (30.6)	13 (13.3)	4 (4.1)	4 (4.1)	2 (2.0)	98 (100.0)
Total	30 (4.2)	12 (1.7)	111 (15.4)	175 (24.3)	205 (28.4)	93 (12.9)	58 (8.0)	30 (4.2)	7 (1.0)	721 (100.0)

## Discussion

AIDS-related deaths are declining worldwide, with the number of AIDS-related deaths decreasing by 39% since 2010 [6]. An increase of more than twice was observed in Turkey between 2009 and 2018. This increase can be interpreted as a real increase considering no change in the death reporting system or HIV/AIDS surveillance during this process. Moreover, the number of new diagnoses in the ECDC Central Europe Region, where Turkey is located, has been reported to increase faster than all other regions [3, 7]. However, much information showing the situation in Turkey in UNAIDS and ECDC reports, which are important for HIV/AIDS, cannot be presented due to the lack of data [3, 4]. The ECDC 2019 report states that the total number of AIDS-related deaths in Turkey in 2009–2018 is 115, and the Ministry of Health provides the source of this information. However, it was found in this study in which the data of the national death registration system are analysed that there were 721 deaths in the same period. In other words, there is a big difference between the data of the Ministry of Health and the data of the death registration system. In the ECDC 2019 report, it is stated that HIV/AIDS reports and death registration system data do not match, and this should be taken into consideration [3]. The Ministry of Health data, which is incompatible with the actual situation regarding HIV/AIDS in Turkey, has been shown in some previous studies, and it has been emphasised that HIV/AIDS records should be improved [8]. The Ministry of Health was asked to provide information about AIDS-related deaths via e-mail before this research. The Ministry of Health replied that, ‘… we still do not have a surveillance system for reported cases, treatment and death data are not collected in our surveillance system within this scope; therefore, we regret to inform you that we cannot meet your data request.’

AIDS-related deaths were 4.5 times higher in men than in women, according to the study. This was thought to be related to the fact that HIV infection is more common in men in Turkey. Previous studies showed that the male/female ratio among people diagnosed varies between 4.4 and 6.2 [9–11]. This significant difference in the number of new diagnoses and AIDS-related deaths in males suggests the ‘men who have sex with men’ cases. Still, cases reported heterosexual relationships more frequently (35.9%), and transmission as a result of homosexual relationships was found to be less frequent (13.3%) in studies conducted [10]. This may be because relationships other than heterosexual relationships are taboo in Turkey, and people do not reveal homosexual relationships as a means of transmission.

In all, 10.2% of AIDS-related deaths in Turkey consisted of people under the age of 15 in 2018. Thirteen per cent of AIDS-related deaths worldwide occurred in people under 15 in the same year [4]. However, there was a significant increase in Turkey in 2018 compared to previous years.

Further studies should be conducted with a biobehavioural survey method specific to risk groups to understand why AIDS-related deaths in Turkey are significantly higher in men or why there are more deaths under the age of 15 in 2018 than in previous years [12].

In conclusion, AIDS-related deaths were increased in Turkey in the 10 years between 2009 and 2018. According to the national death registration system, the number of deaths is much higher than the data from the Ministry of Health. Establishing a strong and accurate HIV/AIDS reporting system and identifying the causes and risk groups of this increase in AIDS-related deaths are critical.

Necessary permissions and ethics committee approval were obtained from the Dokuz Eylul University School of Medicine Ethics Committee (Protocol Number: 2021/04-42) before the research.

## Data

The datasets generated during and/or analysed during the current study are available from the author on reasonable request.
